# Lidocaine-Prilocaine Cream as Analgesia for IUD Insertion: A Prospective, Randomized, Controlled, Triple Blinded Study

**DOI:** 10.5539/gjhs.v7n4p399

**Published:** 2015-01-20

**Authors:** Samira Tavakolian, Mahbobeh Ahmadi Doulabi, Alireza Akbarzade Baghban, Alireza Mortazavi, Maryam Ghorbani

**Affiliations:** 1 Shahid Beheshti University of Medical Sciences, Tehran, IR Iran; 2 Department of Midwifery, Shahid Beheshti University of Medical Sciences, Tehran, IR Iran; 3 Department of Biostatistics, Faculty of Rehabilitation Sciences, Shahid Beheshti University of Medical Sciences, Tehran, IR Iran; 4 Department of Pharmaceutics, School of Pharmacy, Shahid Beheshti University of Medical Sciences, Tehran, IR Iran

**Keywords:** Lidocaine, Prilocaine, IUD, pain

## Abstract

**Introduction:**

Copper IUD is a long term and reversible contraception which equals tubal ligation in terms of sterilization. One of the barriers to using this contraception method is the fear and the pain associated with its insertion. Eutectic mixture of local anesthetics (EMLA) 5% is a local anesthetic that contains 25 mg lidocaine and 25 mg of prilocaine per gram. Application of topical analgesic cream to the cervix for laser surgery, hysteroscopy and hysterosalpingography is known

**Aims:**

this study aimed to determine the effect of EMLA on IUD insertion pain.

**Methods:**

This triple blind clinical trial was conducted on 92 women in a clinic in Hamedan in 2012. After applying the cream on the cervix, pain in three steps, after using Tenaculum, after inserting hystrometr and after inserting IUD and removing IUD insertion tube were assessed with visual analog scale and were compared in EMLA group and placebo group

Statistical analysis used to determine and compare the pain of independent t tests, Mann-Whitney U test and repeated measures analysis of variance and chi-square tests to determine the homogeneity of variables and Fisher’s exact test was used

**Results:**

Insertion hystrometr was determined as the most painful IUD insertion. The mean pain at step 2 (inserting hystrometr) was (3/11±2/53) in EMLA group, (5/23±2/31) in placebo group. EMLA cream significantly reduced the pain after using tenaculum (P<0/001), pain inserting Hystrometr (P< 0/001) and pain at IUD insertion and removing insertion tube (P< 0/001)

**Conclusions:**

Topical Application of EMLA 5% cream as a topical anesthetic on the cervix before insertion IUD reduced the pain during this procedure.

## 1. Introduction

Copper IUD is a long term and reversible contraception which equals tubal ligation in terms of sterilization (Berek & Novak, 2007, WHO/RHR, 2008). One of the barriers to using this contraception method is the fear and the pain associated with its insertion (Murty, 2003). The mucosal lining of female genitalia is extremely sensitive to pain and most small procedures in that area are performed without analgesia (Rodney et al., 1992, De Laco, Marabini, Stefanetti, Del Vecchio, & Bovicelli, 2000). Studies show that about half of the people suffer average to severe IUD insertion pain (Mohammad-Alizadeh-Charandabi, Seidi, & Kazemi, 2010). This feeling varies from a little pain and discomfort to severe cramps with nausea and malaise (Murty, 2003). Since nerve endings are more abundant in cervix particularly internal os than in the body of uterus (Kozman et al., 2001). IUD insertion can incur pain in many different ways including: using tenaculum to hold cervix, using tenaculum to straighten uterus axis, inserting the catheter and IUD insertion tube, and finally inserting IUD. The factors that can aggravate the pain of IUD insertion include nulliparity, age over 30, long interval with pregnancy or last menstrual, and absence of breast feeding (Hubacher, Reyes, Lillo, Zepeda, Chen, & Croxatto, 2006).

If the pain is not relieved, it can increase the risk of vasovagal shock and cardiac arrhythmias (Tolcher, 2003; Berek & Novak, 2007). Studies show that common medication such as uterine cramp reducing medication (Non-steroidal anti-inflammatory drugs), cervix ripening medications (Misoprostol) and local anesthetics (lidocaine) are not effective in reducing IUD insertion pain (Hubacher et al., 2006; Saav et al., 2007; Allen, Bartz, Grimes, Hubacher, & Brien, 2009; Mohammad, Alizadeh, Charandabi et al., 2010).

Eutectic mixture of local anesthetics 5% (EMLA) is a local anesthetic that contains 25 mg lidocaine and 25 mg of prilocaine per gram (Astra Zeneca, 2004). It has been applied locally on cervix for laser surgeries, hysteroscopy, and hysterosalpingography (Zullo et al., 1999; Zilbert, 2002; Liberty et al., 2007).

IUD insertion is a painful process and no globally accepted standard medication is recommended for painless IUD insertion; therefore, this study aimed to determine the effect of EMLA on IUD insertion pain.

## 2. Patients & Methods

This triple blind clinical trial was conducted on 92 women in a clinic in Hamedan in 2012. On the basis on previous studies, standard deviation of 2.55, mean IUD insertion pain score of 3.5 and the sample size of 92 were calculated for this study.

**Figure 1:**
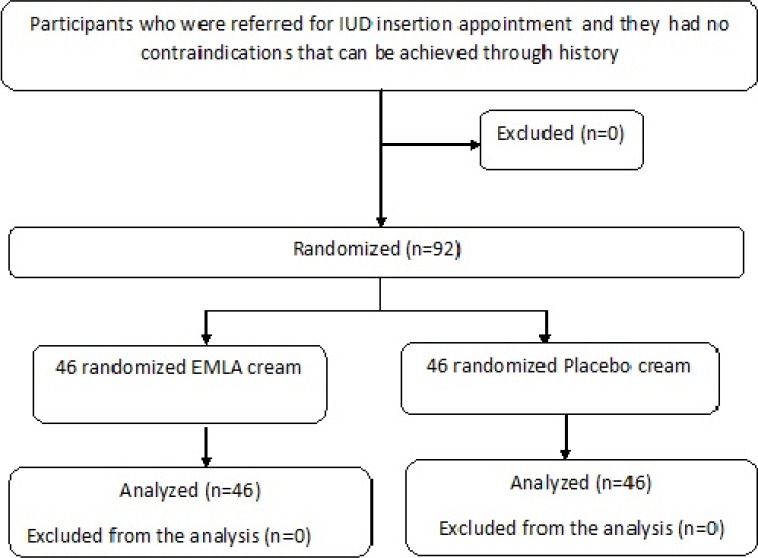
Stratified random sampling was used to allocate the samples in blocks of 4. Most probable confounding variables were controlled, such as age, number and type of delivery. So, people in each block were matched for age, type and number of deliveries

The participants were randomly placed into two groups of 46 as the EMLA and the placebo groups [Figure 1].

The inclusion criteria were absence of contraindication for IUD insertion, not taking analgesics (acetaminophen, ibuprofen, mefenamic acid) 6h before admission, absence of sedative use 24 h before admission, no history of severe mental stress in the past two months. The exclusion criteria included allergy to EMLA (pruritus, burning sensation, edema in cervix), uterus size less than 6 cm and more than 9 cm, and cervix stenosis.

Data collection tool was a researcher-made questionnaire for recording personal information, obstetrics history, observation checklist and 10-cm Visual Analog Scale (VAS). This scale is a 10-cm line graded from 0 to 10, where, 0 means no pain and 10 shows the most severe pain an individual can experience. VAS is rated as 0 for no pain, 1-3 for mild pain, 4-6 for average pain, and 7-9 for severe pain and 10 for extremely severe pain. This scale has been validated in many different studies and has an appropriate validity and reliability (Barker et al., 2006; Fassoulaki, Paraskeva, Patris, Pourgiezi, & Kostopanagiotou, 2003; Wall & Melzack, 2006; Liberty et al., 2007, Shahrjerdi, 2010; Mohammad-Alizadeh-Charandabi et al., 2010) Content validity was used to validate obstetrics questionnaire.

The EMLA cream (5 g tube) used in this study contained 25 mg lidocaine and 25 prilocaine (Astara Zeneca, Sweden, Batch number 003021), and the placebo was locally made by the laboratory of Pharmacy Faculty of Shahid Beheshti University of Medical Sciences and was identical to EMLA cream in terms of appearance, viscosity, color and smell. In order for blinding, the creams were packaged in similar sterile containers and were given to the researcher.

The women requesting to have IUD inserted, meeting the inclusion criteria and agreeing to participate in the study were enrolled. Then they were informed of the objectives and groups of the study and signed the written consent.

First, they lay in lithotomy position and their vulva, vagina and cervix were examined for inflammation, abnormal findings and secretions. Bimanual examination of uterus was performed to investigate its size, position and consistency. After rinsing vagina and cervix with betadine twice, 5 g of the cream was applied to the cervix and external os using a cotton swab. The researcher and the patients were blind to the type of the cream. Seven minutes was allocated for the onset of anesthesia. Then the severity of pain was measured using VAS in 3 stages of inserting IUD: stage 1 (using the tenaculum), stage 2 (after inserting hysterometer) and stage 3 (after inserting IUD and removing IUD insertion tube). The data were analyzed with SPSS 17 using descriptive and inferential statistics. The data were entered before the type of cream was determined. In order to study the homogeneity of the two groups in terms of background information or possible confounding variables, Chi square and Fisher’s exact test were used. Quantitative variables were compared by using independent t test if quantitative variables were distributed normally and by using non-parametric Mann-Whitney test if they were not distributed normally. For univariate within subject analysis repeated measures analysis variance was used. P value less than 0.05 was considered significant.

## 3. Results

Majority of the participants (37% in the placebo group and 45.7% in the EMLA group) were referred in the third day of menstruation. Mann-Whitney and Fisher’s exact test and Chi square test showed that the two groups were matched for personal factors like age, job, spouse’s job, education, and obstetrics variables like menstrual period day, number of pregnancies, type of delivery, number of deliveries, breast feeding, history of inserting IUD and history of cryo (Table 1).

**Table 1:** Demographic and obstetrics variables by the groups ^*^Using Mann-Whitney, ^**^Using Chi square test

	Placebo group (n=46)	EMLA group (n=46)	P
Age (Years)	26/43±4/52	26/78±4/07	0.66^*^
Number of pregnancies	1/93±0/989	2/09±1/007	0.45^*^
Number of deliveries	1/74±0/874	1/78±0/786	0.74*
Past vaginal delivery (%)	24 (52/2)	*24 (52/2)*	1.0^**^
Past Cesarean Section (%)	22 (47/8)	22 (47/8)	1.0^**^
Breast feeding (%)	29 (63)	29 (63)	0.85^**^
History of inserting IUD(%)	15 (32/6)	(32/6)15	1.0^**^
History of cryo (%)	0	*2 (4/3)*	0.9^**^

The results of the statistical analysis showed that most study units in the EMLA group (50%) were pain-free at the stage of using tenaculum and 39% in the placebo group had mild pain. During the insertion of hysterometer, most study units in the EMLA group (39.1%) experienced mild pain while 47.8% in the placebo group suffered from average pain. During IUD insertion and removing insertion tube, most study units in the EMLA group (41.3%) and the placebo groups (41.3%) reported mild pain.

**Figure 2:**
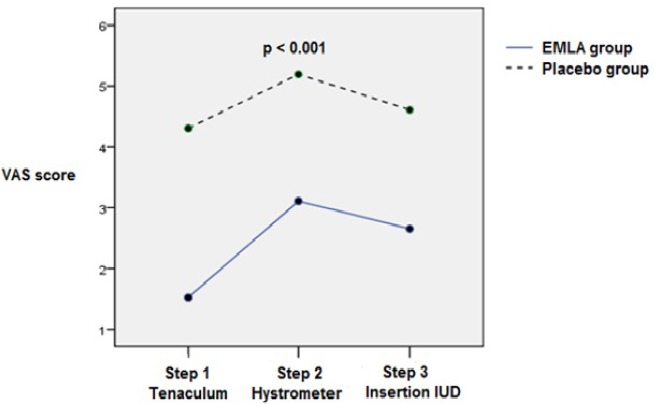
The severity of pain was measured in 3 stages

The severity of pain was measured in 3 stages. The mean of pain during the first stage (using tenaculum) was 4.30±2.40 in the placebo group and 1.52±1.85 in the EMLA group, and the difference was significant (P<0.001). The second stage (inserting hysterometer) was recognized as the most painful stage (P<0.001) (Figure 2).

The mean pain during the second stage (inserting hysterometer) was 5.20±2.31 in the placebo group and 3.11±2.53 in the EMLA group, which showed a significant difference (P<0.001). There was a significant difference between the two groups in the third stage (inserting IUD and removing the insertion tube) as it was 4.61±2.55 in the placebo group and 2.65±2.53 in the EMLA group (P<0.001).

## 4. Discussion

Since IUD is an acceptable contraception, many studies have been conducted on the IUD insertion pain. Most studies have investigated the effect of chemical drugs including NSAIDs (naproxen, ibuprofen) and cervix ripening medications (misoprostol), and showed their neutral effect in this regard (Hubacher et al., 2006; Saav et al., 2007). The results of these studies showed that IUD insertion pain in not caused by prostaglandin secretion. Few studies have been conducted on applying local anesthetics on IUD insertion pain. One such study is Naldenberg’s in 2007, which examined the effect of paracervical method on IUD insertion pain. He concluded that although this invasive method reduced IUD insertion pain, it had complications like tremor/chills, and that its toxicity can cause complications like hypotension, bradycardia, audiovisual disorders, lightheadedness, muscular contraction, seizure, coma, apnea, and cardiovascular arrest (Nadelberg, 2007). Alizadeh et al. studied the effect of lidocaine gel on IUD insertion pain in Tabriz in 2011 and found no significant difference between the lidocaine gel group and placebo group in terms of IUD insertion pain (De Laco et al., 2000). The reasons behind this finding can be use of little amount of anesthetic (1 ml), little time of application of the gel on the cervix before procedure (1 min) and lack of variety of anesthetics. The present study investigated the effect of EMLA cream on cervix in 3 stages of inserting IUD. The results showed that inserting hysterometer in cevix is the most painful stage and those who received EMLA cream had less pain as compared with those who received placebo (p<0.001). Furthermore, EMLA cream caused less pain in 3 stages of using tenaculum, inserting hysterometer and inserting IUD and removing insertion tube (p<0.001). These findings are in line with those of Liberty et al. in 2007 on the effect of EMLA on hysterosalpingography pain (Liberty et al., 2007) and those of Stiglano et al. in 1997 on the effect of EMLA on hysteroscopy pain and those of Zibert in 2002 on the effect of EMLA on laser induced pain in cervix (Stiglano et al., 1997; Zibert, 2002). Liberty et al., recognized manipulating cervix as the most painful stage. They also. eported a significant reduction in pain by applying EMLA cream at cervix manipulation stage (Liberty et al., 2007). Pain transmission from uterus and cervix occur in two different pathways. Sensory fibers go to the central nervous system through T11 and T12 nerve roots while sensory fibers of cervix go through the 2^nd^, 3^rd^, and 4^th^ sacral nerves. The possible mechanism of EMLA cream is blocking the pain in the 2^nd^, 3^rd^ and 4^th^ sacral nerves. This mechanism has been confirmed by Liberty et al., (2007).

## 5. Conclusion

The results of present study shows that topical Application of EMLA 5% cream as a topical anesthetic on the cervix before insertion IUD reduced the pain during this procedure. Since inserting IUD is painful and involves cervix canal and environment, applying EMLA cream on cervix can significantly reduce pain through the possible mechanism of blocking the 2^nd^, 3^rd^ and 4^th^ sacral nerve
